# Varicella and Zoster Vaccination Strategies in Immunosuppressed Pediatric Transplant Recipients

**DOI:** 10.3390/vaccines13050534

**Published:** 2025-05-16

**Authors:** Christopher Hartley, Priscila Villalba Davila, Emma Cole, Wikrom Karnsakul

**Affiliations:** 1 The Department of Pharmacy, The Johns Hopkins Hospital, Baltimore, MD 21287, USA; 2 Division of Pediatric Gastroenterology, Hepatology and Nutrition, Department of Pediatrics, Johns Hopkins University of Medicine, Baltimore, MD 21287, USAwarnsa1@jhmi.edu (W.K.)

**Keywords:** varicella vaccine, pediatric transplantation, immunosuppression, vaccine safety, live-attenuated vaccines, post-exposure prophylaxis

## Abstract

The varicella vaccine has prevented varicella in hundreds of thousands of patients since its establishment in 1974. It stimulates both humoral and cell-mediated immunity to produce an immune response that helps protect against the disease (not necessarily the infection). Serious sequala of varicella including pneumonia, hepatitis, and encephalitis can occur, with higher incidence in immunosuppressed individuals than in the general population. Patients who are not immunosuppressed should receive routine varicella vaccinations. For those who have not completed the series or are significantly distant from their last immunization, serologic testing may be considered. In pre-transplant patients, live-attenuated vaccines should ideally be administered at least four weeks before transplantation. Case studies have documented instances of patients requiring treatment for varicella after receiving a transplant within four weeks of vaccination.

## 1. Introduction

Varicella or chickenpox, caused by the varicella-zoster virus (VZV), is the primary infection, with the virus potentially reactivating later in life as herpes zoster (shingles). Vaccination is, thus, crucial for preventing both conditions [1,2]. During the pre-vaccine era, varicella was a major public health concern in the United States, with an estimated 4 million cases, 11,000–13,500 hospitalizations, and 100–150 deaths per year [1]. The introduction of a single-dose vaccine in 1995 led to substantial declines in varicella cases and associated health problems, and the addition of a second dose further improved outcomes, resulting in a more than 97% reduction in incidence and reductions of over 90% in varicella-related hospitalizations and deaths [1,3,4].

The first live-attenuated varicella vaccine was developed in Japan by Dr. Michiaki Takahashi in 1974. This vaccine, known as the Oka strain, was derived from the vesicular fluid of a child with varicella and was found to be safe and effective in preventing varicella. However, this vaccine is neurovirulent and can cause VZV to become latent and later reactivate as herpes zoster (shingles) [5]. Most recently, a recombinant subunit vaccine targeting the VZV glycoprotein E has been developed. This vaccine has shown high efficacy in preventing herpes zoster and post-herpetic neuralgia in adults by producing a robust immune response without risking viral reactivation, making it a safer option for immunocompromised patients [6]. However, there are no existing guidelines or evidence supporting the use of this vaccine in pediatric patients. It is approved for adults aged 50 and older, as well as for adults aged 18 and older who are at a higher risk of herpes zoster due to immunodeficiency or immunosuppression from a known condition or treatment [7]. Patients receive this recombinant subunit vaccine (Shingrix^®^) if they have had varicella prior (either from vaccination or from disease). Patients who are seronegative for varicella would instead need to receive a live-attenuated vaccine, such as Biken^®^, Varilrix^®^, or Varivax^®^.

Post-transplant pediatric patients with a seronegative status for VZV at the time of transplant have a high risk of developing primary varicella infection and VZV reactivation. These patients can also develop severe cases of disseminated varicella, which can affect multiple organs and lead to complications such as encephalitis, pneumonitis, and hepatitis [8,9]. Donor-transmitted VZV infection is rare but has previously been reported. The first published case involved a 15-month-old girl who developed a primary VZV infection following cardiac transplantation from a donor who had been treated for primary varicella two weeks before the donor’s death [10].

VZV seropositivity varies significantly by region, age, and vaccination coverage. According to the data from the National Health and Nutrition Examination Survey (NHANES) conducted between 1999 and 2004, in the United States, the VZV seroprevalence among children aged 6–19 years was approximately 93.6%. This high seroprevalence is indicative of the widespread immunity achieved through both natural infection and vaccination efforts [11]. In immunosuppressed pediatric transplant recipients, the seroprevalence rates are lower. Seronegativity ranges from 7% to as high as 50% in these patients. This variability is influenced by factors such as the recipient’s age, prior vaccination status, and pre-transplant immunosuppression [8]. This is particularly concerning given the high morbidity and mortality of disseminated VZV in immunocompromised hosts. Thus, pre-transplant serologic screening is essential for risk stratification and consideration of prophylactic or preemptive strategies. The American Society of Transplantation emphasizes the importance of such screening and the implementation of appropriate preventive measures [8].

This review will discuss the current existing knowledge gaps surrounding varicella and herpes zoster, as well as clinician hesitancy in administering live vaccines in immunosuppressed pediatric transplant recipients. Ultimately, it aims to inform improved strategies for protecting this high-risk population.

## 2. Vaccine Efficacy and Safety in Pediatric Transplant Patients

Guidelines from the Infectious Diseases Society of America (IDSA, 2013) and the American Society of Transplantation Infectious Diseases Community of Practice (AST IDCOP, 2019) recommend varicella vaccination for select patients on minimal immunosuppression who are at risk of severe disease if infected. Studies assessing live varicella vaccine administration in post-transplant patients have reported variable but generally favorable outcomes, with seroconversion rates ranging from 25% to 100% depending on patient characteristics and immunosuppressive regimens. The guidelines provide guidance on certain individuals on minimal immunosuppression who would be considered at high risk of a negative outcome from varicella if infected [9,12].

A summary of key studies (Table 1) highlights the safety and efficacy of live varicella vaccination in transplant recipients [13]. In a review by Danerseau, only one case of organ rejection was reported post-vaccination, and vaccine-associated varicella occurred at a rate consistent with that in immunocompetent individuals [13]. Similarly, Feldman et al. observed high seroconversion rates post-vaccination, with no reported cases of organ rejection or severe adverse effects [14]. Kano et al. further demonstrated sustained immunity up to three years post-vaccination [15]. These findings suggest that, in carefully selected transplant recipients, varicella vaccination may be both effective and well tolerated.

### 2.1. Immunological Considerations

Evidence of immunity to varicella can be determined through several criteria for the pre-transplant evaluation for VZV immunity. One way is by documenting age-appropriate immunization. For preschool-aged children (12 months to 3 years), one dose of the varicella vaccine is required, while school-aged children, adolescents, and adults need two doses to be considered adequately vaccinated. The varicella vaccine is normally administered at least 3 months after the first dose but can be administered as early as 4 weeks after the previous dose. Another way to verify immunity to VZV is through laboratory evidence, such as a laboratory-confirmed history of varicella or zoster. Additionally, birth in the United States before 1980 may indicate presumed immunity to VZV, though this is not applicable for healthcare personnel, pregnant individuals, or those who are immunocompromised. Lastly, a history of varicella or zoster confirmed by a healthcare provider can serve as evidence of prior infection and indicate prevention of the disease [19].

The varicella-zoster virus (VZV) vaccine stimulates both humoral and cell-mediated immune responses, which in turn help produce an immune response to protect against varicella infection. Studies have demonstrated a correlation between vaccine-induced antibody production and immunity. However, in healthy individuals, protection from varicella often persists even after antibody levels decline to undetectable levels, suggesting that CD4+ T cell responses play a critical role in long-term protection. As a result, the presence of anti-VZV antibodies may primarily serve as an indirect marker of protective cellular immunity rather than being the sole determinant of immunity [20,21,22]. Natural-killer cells also serve a critical role in immunity to help protect against disease. In patients with natural-killer cell deficiencies, the risk of fatal herpesvirus infections is much higher [23].

Varicella infection in immunocompromised patients can lead to serious complications such as pneumonia, hepatitis, and encephalitis. Therefore, documenting immunity through vaccination is strongly recommended for transplant candidates, as feasible. However, the use of live viral vaccines in post-transplant patients remains controversial due to concerns about safety and efficacy. A 2008 systematic review examining the post-transplant administration of attenuated live vaccines found that 57% of patients achieved immune-range titers after a single dose of either the MMR or varicella vaccine, with seroconversion rates ranging from 41% to 94%. Despite this, all the studies showed a decline in titers over time. Out of 89 patients vaccinated for varicella, 3 developed mild disease, likely from community-acquired infection [16]. In a pediatric cohort of 16 liver or intestinal transplant recipients, 87% demonstrated immunity at 12 weeks post-transplant [18]. Another study of 17 pediatric kidney transplant recipients showed a peak immunity rate of 94%, which decreased to 35% by 4 years [24]. A recent review of 16 studies involving 339 solid organ transplant recipients who received live viral vaccinations found seroconversion rates for varicella ranging from 25% to 87%, with some variation depending on immunosuppressive regimens. Generally, the seroconversion rates for measles, mumps, and rubella vaccines are higher than that for varicella [25]. Although live viral vaccines may induce seroconversion after transplantation, immunity can diminish over time. Thus, booster doses may be necessary to maintain protective antibody levels, even while patients are on immunosuppression.

Immunosuppressive regimens, while essential for graft survival, significantly impair both humoral and cellular responses to vaccination. Agents such as corticosteroids, calcineurin inhibitors, and antimetabolites like mycophenolate mofetil reduce T and B cell proliferation, blunt antigen presentation, and interfere with memory formation. Consequently, the vaccine-induced seroconversion rates are expected lower, and the duration of protection is shorter in transplant recipients than in immunocompetent individuals. The timing of vaccination relative to immunosuppression intensity, as well as drug-specific effects, must be considered when devising immunization strategies [14].

#### 2.1.1. Role and Dilemma of Serologic Testing Pre- and Post-Vaccination

Serologic testing plays a critical role in assessing immune responses to varicella vaccination both before and after immunization. The current guidelines recommend two doses of the varicella vaccine for VZV-naïve patients to establish and maintain immunity. However, in some cases, an additional third dose may be necessary to achieve a sufficient immune response. In one study, 21.9% (7/32) of children required a third dose, which led to a significant increase in anti-VZV antibody titers [17]. This rate of nonresponse after two doses appears to be higher than that observed in healthy children. Although antibody titers serve as a useful marker of varicella immunity, they may not fully capture vaccine-induced protection due to the critical role of CD4+ T cells in the immune response to VZV. The durability of this serologic response was notable, with no breakthrough varicella cases reported over four years of follow-up, despite frequent potential exposures in an unvaccinated community setting. T cell-mediated immunity is believed to play a key role in varicella protection. For example, individuals with agammaglobulinemia often experience uncomplicated varicella, whereas those with T cell deficiencies face a higher risk of severe complications. While VZV-specific T cells have been detected in immunized pediatric liver transplant recipients, their precise role in long-term immunity remains unclear. Notably, higher frequencies of VZV-specific CD4+ T cells have been observed following natural varicella infection compared with those after vaccination, likely reflecting the stronger immune stimulation from wild-type VZV [17]. Post-transplant varicella vaccination has also been associated with a measurable increase in VZV-specific T cell responses. However, interpreting the clinical significance of these T cell responses remains challenging, as no established protective threshold exists. The extended duration of protection in healthy individuals despite declining antibody levels suggests a key role for T cell-mediated immunity, but whether a more robust T cell response translates to prolonged immunity is still uncertain. Further research is needed to clarify the relationship between T cell responses, antibody titers, and long-term vaccine efficacy in immunosuppressed populations.

#### 2.1.2. Timing of Vaccination in Relation to Transplantation and Immunosuppression

The timing of live vaccination is crucial in transplant candidates and recipients due to the risk of vaccine-derived varicella occurring too close to transplantation. The AST-IDCOP 2019 guidelines recommend administering live-attenuated vaccines, including varicella and measles–mumps–rubella (MMR), at least four weeks before transplant [9]. This allows sufficient time for viral replication and antibody development, minimizing the risk of vaccine-related complications post-transplant. However, the unpredictability of transplant timing poses a challenge. Patients listed for transplantation may receive an organ offer at any time, making it difficult to adhere to strict pre-transplant vaccination schedules. The transplant team must carefully weigh the risks and benefits of live vaccination, particularly in patients with urgent transplant needs. In cases in which vaccination is not feasible pre-transplant, alternative strategies such as post-exposure prophylaxis and antiviral therapy may be considered to mitigate varicella risk.

Historically, live-attenuated vaccines such as the measles–mumps–rubella (MMR) and varicella (VZV) vaccines were contraindicated in solid organ transplant (SOT) recipients due to fears of vaccine-associated disease under immunosuppressive therapy. However, growing evidence supports their safety and immunogenicity in carefully selected pediatric transplant recipients. A recent multicenter U.S. cohort study of 281 pediatric liver and kidney transplant recipients demonstrated that post-transplant live viral vaccination was well tolerated, with no vaccine-related graft injuries or deaths. Among those vaccinated, protective antibody responses were achieved in 72% for VZV and in over 83% for each MMR component, with sustained immunity at 1 year post-vaccination in the majority of responders. Notably, a subset of patients who fell outside of the traditional eligibility criteria—including those receiving mycophenolate mofetil, exhibiting detectable Epstein–Barr virus (EBV) DNA, or classified as being under moderate immunosuppression—also achieved protective titers without severe adverse events. These findings suggest that the strict criteria traditionally endorsed by the American Society of Transplantation and Suresh et al. (e.g., >12 months post-transplant, minimal immunosuppression, adequate lymphocyte counts) may warrant reevaluation [26]. Mild post-vaccination adverse events such as transient lymphadenopathy and self-limited breakthrough varicella occurred in a small number of patients, exclusively among those with moderate or higher immunosuppression. Importantly, no episodes of acute rejection were observed within one month of vaccination. Collectively, these data advocate for a nuanced, risk-adapted approach to live vaccination post-transplant that balances safety, immunogenicity, and the high disease burden of VZV in this population [14].

### 2.2. Global and Regional Guidelines

According to the Centers for Disease Control (CDC) immunization schedule by medical indication, patients who are immunosuppressed (excluding those with human immunodeficiency virus), the varicella vaccine is contraindicated and not recommended [27]. Within the transplant population, many primary care providers request information from the patient’s transplant team regarding vaccine schedule, and many transplant clinicians defer live-attenuated vaccines. The largest bodies of guidance in this area include the 2013 Infectious Diseases Society of America (IDSA) on vaccination in the immunocompromised host and the 2019 American Society of Transplantation Infectious Diseases Community of Practice (AST-IDCOP) guideline on the vaccination of solid organ transplant candidates and recipients. Both guidelines lack strong recommendations on whom to vaccinate [9,12]. The 2013 IDSA recommendation was to not give live vaccines post-transplant, whereas the 2019 guideline suggests live vaccines, such as varicella, could be safe and effective for certain “at-risk” populations.

## 3. Practical Barriers to Vaccination

Many transplant clinics do not administer routine vaccinations, instead referring patients to their primary care providers. This creates a barrier within the healthcare system, as primary care providers often require specific guidance on the safety of vaccines—particularly live-attenuated vaccines—in immunocompromised individuals. Since they typically consult the patient’s transplant team before proceeding, this can lead to delays in vaccination, leaving patients in a “catch-up” phase and potentially unprotected for extended periods.

A more integrated approach, in which transplant clinics administer vaccines directly and discuss risks in real time with the team managing immunosuppression, could streamline the process and improve adherence. The lack of standardized post-transplant vaccination protocols further underscores the need for transplant centers to take a more active role in vaccination.

Currently, in the United States, the only post-transplant vaccination mandated by the United Network for Organ Sharing (UNOS) is the annual inactivated trivalent influenza vaccine. Beyond this, patient-specific factors must be carefully considered when determining the appropriate timing for vaccination. These include the patient’s immune status, serology results, and any contraindications that may impact vaccine efficacy or safety.

## 4. Post-Exposure Prophylaxis (PEP)

Solid organ transplant (SOT) recipients face significant risks from varicella-zoster virus (VZV) exposure due to immunosuppression. Exposure can occur via several mechanisms [28].

1. Direct Exposure: Transmission occurs through contact with symptomatic individuals. Hospitalized varicella patients require airborne and contact precautions until lesions crust over (typically ≥5 days). Immunocompromised patients may require extended isolation. Breakthrough varicella, even in vaccinated individuals, warrants continued precautions until no new lesions appear for 24 h. Exposed, nonimmune individuals should follow precautions for 8–21 days post-exposure, extending to 28 days if treated with varicella-zoster immune globulin (VZIG).
2. Asymptomatic Shedding and Indirect Exposure: VZV can spread via respiratory droplets or vesicular fluid, even in individuals with mild or unrecognized reactivation.
3. Reactivation of Latent VZV: Immunosuppression increases the risk of herpes zoster (shingles) due to viral reactivation from the dorsal root ganglia.
4. Household Exposure: Varicella vaccination in household contacts carries minimal risk of transmission, but the benefits of preventing severe varicella in immunocompromised individuals outweigh this concern.

Post-exposure prophylaxis (PEP) strategies, including passive immunization with varicella-zoster immune globulin (VZIG) and antiviral therapy, are essential in preventing severe disease. We evaluate the current evidence and guidelines on PEP for VZV exposure in pediatric transplant recipients, emphasizing efficacy, timing, and clinical considerations.

VZV infections can be severe and can cause life-threatening complications in pediatric solid organ transplant (SOT) recipients due to their immunosuppressed status. While vaccination is a primary strategy for prevention, many SOT recipients are nonimmune at the time of transplantation, requiring effective post-exposure prophylaxis (PEP) following contact with a person with a known varicella or zoster infection. It is essential to consider the degree and type of immunosuppression when determining the need for VZIG administration, and consultation with pediatric infectious disease or immunology specialists may provide valuable guidance. PEP aims to prevent or mitigate VZV infection in exposed, susceptible patients using passive immunization with varicella-zoster immune globulin (VZIG) and/or antiviral therapy such as acyclovir or valacyclovir.

VZIG provides passive immunity by supplying preformed anti-VZV antibodies, reducing the severity and complications of infection. Intramuscular (IM) injection is the standard route of administration and should be administered within 96 h of exposure (preferably as soon as possible) for optimal effectiveness. The dose may be considered beyond 96 h in cases of high-risk exposure, although the efficacy declines. Age-banded standardized dosing is utilized in pediatric patients for VZIG, and adults receive 625 IU for their dose. Each vial of VZIG is 125 IU/vial. Refer to Table 2 for VZIG dosing in pediatric patients [29]. Of note, VZIG reduces the risk of severe varicella but does not completely prevent infection. Patients who develop breakthrough varicella after VZIG typically experience attenuated disease with fewer complications. In cases in which patients receive regular high-dose intravenous immunoglobulin (IVIG) therapy (400 mg/kg or more), protection may be provided if the most recent dose was administered within three weeks of exposure. Following VZIG administration, patients should receive age-appropriate varicella vaccination when possible, provided live vaccines are not contraindicated [19].

### Antiviral Therapy (Acyclovir/Valacyclovir)

In cases in which passive immunoprophylaxis is not available, antiviral chemoprophylaxis may be used. Antiviral agents inhibit VZV replication, reducing viral load and disease severity. Its indication is an adjunct to VZIG in high-risk patients. For PEP beyond 96 h post-exposure when VZIG is unavailable, acyclovir (oral or IV) or valacyclovir (oral) is typically given for 7–10 days starting 7 days post-exposure. IV acyclovir is preferred for severely immunosuppressed patients. Antiviral prophylaxis significantly reduces the risk of severe varicella in transplant recipients. This, however, may be used alone when VZIG is unavailable or impractical. Valacyclovir (20 mg/kg per dose, administered orally three times daily, with a maximum daily dose of 3000 mg) or acyclovir (20 mg/kg per dose, administered orally three times daily, with a maximum daily dose of 3200 mg) can be administered as post-exposure prophylaxis for immunocompromised patients lacking immunity or for immunocompetent patients seeking varicella prevention. Antiviral treatment should begin 7 days after exposure and continue for 7 days for immunocompromised patients. For VZV-seropositive patients undergoing intensive or myeloablative chemotherapy, antiviral prophylaxis is recommended. However, children receiving antiviral treatment with valganciclovir, ganciclovir, or foscarnet do not require additional VZV prophylaxis.

The clinical considerations in PEP include timing and risk assessment. Early intervention is critical as PEP should be initiated as soon as possible after exposure. PEP for high-risk patients, such as those who have received recent treatment for organ rejection with high-dose steroids and or lymphocyte-depleting agents, requires VZIG + antiviral therapy. Lower-risk patients with stable immunosuppression should receive either VZIG or antivirals alone. Breakthrough infection may still occur despite PEP, but it is typically milder.

Symptoms (fever and rash) should be monitored for 21 days post-exposure. Prompt antiviral therapy initiation is recommended if breakthrough varicella occurs. Preemptive screening for VZV immunity in transplant candidates may help guide PEP strategies. A policy such as hospital infection control measures to isolate exposed, nonimmune transplant recipients should be implemented to prevent nosocomial transmission. Further studies should include efficacy studies comparing VZIG vs. antivirals alone in transplant recipients as well as alternative prophylactic strategies, including the potential use of recombinant zoster vaccine in seronegative SOT recipients. Antiviral dosing for maximum effectiveness should be considered in pediatric transplant patients.

In summary, PEP is essential for preventing severe varicella-zoster virus infection in pediatric solid organ transplant recipients. VZIG remains the standard of care, with antiviral therapy serving as an important adjunct or alternative in certain cases. Timely risk assessment and individualized prophylaxis strategies are critical to ensuring optimal outcomes in this high-risk population.

## 5. Varicella Vaccine Innovations and Future Directions

The recombinant zoster vaccine (RZV), known as Shingrix, has significantly advanced the prevention of herpes zoster (HZ), especially among immunocompromised adults, covering immunocompromised individuals over 18. RZV is the first and only HZ vaccine approved for use in immunocompromised adults globally, including in Europe and the U.S. The Advisory Committee on Immunization Practices (ACIP) recommends a two-dose regimen of RZV for immunocompromised adults aged 19 and older based on evidence suggesting that RZV restores levels of anti-varicella-zoster virus cellular and humoral immunity. These levels are often diminished in immunocompromised adult patients [30]. However, its use in the immunocompromised pediatric population has not been extensively studied; therefore, the current guidelines do not recommend RZV for this group. There is utility in promoting future research to determine whether recombinant vaccines, like RZV, could be adjusted for use within the pediatric transplant population.

For pediatric patients, the varicella vaccine is recommended to prevent primary infection from the virus. Studies have demonstrated that the varicella vaccine is immunogenic and clinically effective in both healthy and immunocompromised children. Historically, when considering cases of severe immunocompromise such as solid organ transplant, vaccination post-transplant should be managed with expert guidance from medical professionals. The ACIP does not recommend administering live vaccines, such as MMR or varicella, to immunocompromised-status patients (excluding those with human immunodeficiency virus), stating that it is contraindicated to vaccinate these individuals [27]. Severe varicella infections in transplant recipients are of significant concern, and vaccinating these patients could help reduce morbidity and mortality [31]. The current guidelines recommend immunizing immunocompetent children routinely against varicella and relying on the resulting herd immunity to protect those who are immunocompromised [32]. Persons who live with immunocompromised individuals within the household are highly recommended and encouraged to be vaccinated against varicella.

There is a significant need for future research in this area, particularly focusing on cellular immunity and T cell responses in varicella vaccination. Since the varicella vaccine mimics natural infection, a single dose is generally sufficient to generate a T cell-mediated response [33]. Studies have demonstrated that the vaccine induces VZV-specific T cell immunity in select populations, including children undergoing treatment for leukemia, other malignancies, liver transplantation, post-stem cell transplantation, and HIV [34]. However, the research on the efficacy of varicella vaccination has primarily focused on immunocompromised children with leukemia, with little to no data available on solid organ transplant recipients. Expanding research in this population is critical to understanding long-term immunity and optimizing vaccination strategies.

Assessing VZV-specific immunity in pediatric transplant recipients is an evolving field, with increasing recognition that standard serologic testing may not sufficiently predict protection in immunocompromised individuals. While VZV IgG titers remain widely used, they offer limited insight into cellular immunity, which is critical for controlling viral reactivation. Emerging assays—such as ex vivo and cultured ELISpot, interferon-gamma release assays (IGRAs), and flow cytometry-based T cell proliferation—enable the more precise characterization of memory T cell subsets responding to VZV antigens [35]. These tools have demonstrated that, while most seropositive individuals exhibit measurable T cell responses to key VZV proteins such as gE and IE63, discordance between antibody levels and T cell activity is common. Notably, cultured ELISpot assays may uncover central memory T cell responses not captured by ex vivo assays, reflecting complementary but distinct aspects of immune memory [35,36].

Studies also show that T cell responses decline during early post-transplant immunosuppression, with gradual recovery by 180 days, correlating with reduced immunosuppressive intensity. Interestingly, some pre-transplant patients, especially those with end-stage renal disease, exhibited unexpectedly robust T cell responses, potentially linked to chronic inflammation [35]. While herpes zoster reactivation was not observed in the monitored cohort, the occurrence of HSV reactivations during peak immunosuppression highlights the need for ongoing surveillance. Ultimately, functional immune assays may offer a valuable adjunct to serology for stratifying reactivation risk, guiding prophylaxis, and tailoring immunosuppressive regimens. Further validation in larger cohorts is needed to standardize these approaches and integrate them into routine post-transplant care [35].

## 6. Public Health Implications

Universal varicella vaccination in pediatric patients undergoing transplant is both cost effective and clinically beneficial. It decreases the incidences of both classic and severe cases of varicella, with the latter being associated with high morbidity and mortality in immunocompromised pediatric patients [8,9]. This reduction in disease burden translates to an improved quality of life for patients and a decreased strain on healthcare resources.

There are several studies that have evaluated the cost effectiveness of the varicella vaccine in pediatric liver and kidney transplant recipients. Olson et al. demonstrated that pre-transplant varicella vaccination in children with chronic renal failure resulted in substantial cost savings, with a cost of USD 211 per vaccinated patient compared with USD 1828 in additional medical costs per unvaccinated patient. This cost effectiveness was driven by the reduced need for expensive interventions such as varicella-zoster immunoglobulin (VZIG) prophylaxis and hospitalization for parenteral acyclovir treatment [37]. Feldman et al. highlighted the high incidence and associated costs of vaccine-preventable infections, including varicella, in pediatric solid organ transplant recipients. They found that 15% of solid organ transplant patients are hospitalized for vaccine-preventable infections within five years of transplant—a rate roughly 87 times as high as that for the general population. The authors emphasized the importance of immunizing all transplant candidates and recipients so as to reduce morbidity, mortality, and healthcare costs [38].

Varicella vaccine coverage varies significantly across the globe, influenced by national immunization policies, economic factors, and public health priorities. Some countries have implemented universal vaccination programs, while others have not. In the Asia-Pacific region, for instance, national vaccination rates range from 3% to 97%, largely depending on whether the vaccine is part of the country’s national immunization schedule [39]. Similarly, in Europe, policies differ widely, with twelve countries having adopted universal vaccination as of 2021, while others have refrained due to concerns about cost effectiveness and potential changes in disease patterns (e.g., shifting varicella to older age groups or increasing the incidence of herpes zoster) [40,41].

As of 2021, 44 countries had incorporated the varicella vaccine into their universal vaccination programs [40]. Some of these countries have chosen a two-dose regimen, while others use a single-dose strategy for economic reasons. Research has shown that the two-dose regimen offers broader protection, preventing more mild cases and reducing breakthrough infections. However, countries such as New Zealand, Turkey, Taiwan, and others still opt for the single-dose approach [42].

The United States became the first country to implement a universal varicella vaccination program in 1995. By 2021, Canada and fifteen Latin American countries had also adopted similar regimes. In contrast, no countries in Africa or Southeast Asia have introduced universal varicella vaccination programs due to limited resources and the need to prioritize other infectious diseases with a higher public health burden [40].

## 7. Conclusions

In summary, while live-attenuated varicella vaccination is contraindicated post-transplant, optimizing pre-transplant immunity and implementing antiviral prophylaxis are essential strategies for mitigating varicella-zoster virus (VZV)-related morbidity in immunosuppressed individuals. Interpreting the clinical significance of increased VZV-specific T cells remains challenging, as no definitive protective threshold has been established. The persistence of immunity despite declining antibody levels in healthy individuals highlights the important role of T cell-mediated protection, although the extent to which stronger T cell responses contribute to long-term immunity is still uncertain. While pre-transplant varicella vaccination remains ideal, immunity may wane over time, particularly in individuals undergoing immunosuppressive therapy. Primary or booster immunization appears to be safe, immunogenic, and effective in pediatric solid organ transplant (SOT) recipients. Based on the current literature, we recommend assessing VZV serology one year post-transplant and considering re-immunization for seronegative patients who meet specific clinical criteria [17].

However, there is an urgent need for updated guidelines to address the gaps in the safety data, particularly regarding the long-term effects of vaccination in transplant recipients. Factors such as the effect of dialysis on vaccination efficacy and post-exposure prophylaxis, as well as individual variations in immune responses, reinforce the importance of personalized decision-making for each transplant recipient. These factors should be considered when determining the timing and type of vaccinations. Furthermore, transplant societies should issue more comprehensive guidance on post-transplant vaccination practices, ensuring that transplant centers have a standardized approach to optimize patient outcomes and minimize the risks of vaccine-preventable infections. Future research should prioritize prospective studies evaluating the long-term safety and immunogenicity of VZV vaccination in pediatric transplant recipients, including stratified protocols based on immunosuppression levels and organ type. Comparative effectiveness studies are needed to assess newer subunit zoster vaccines (e.g., recombinant zoster vaccine) in immunocompromised pediatric populations, though currently not licensed for children. In parallel, the development and validation of biomarkers of protective immunity—both humoral and cellular—may allow tailored vaccination schedules and real-time monitoring of immunity. Lastly, registries and international collaborations are critical to improve data collection, harmonize guidelines, and enable broader policy recommendations.

## Figures and Tables

**Table 1:** Studies of live varicella vaccines in post-transplant patients. Adapted from [13].

Study Author	Intervention	Number of Patients	Number of First Vaccine Doses Received	Number of Second Vaccine Doses Received	Number of Third Vaccine Doses Received	Outcome	Median Time Since Transplant in Years (IQR)	Results
Danerseau Review [16]	MMR and Varicella	112	89	7	-	Safety and Efficacy	Multiple smaller case studies	1 case of rejection 3 weeks post-immunization, 9 possible cases. 1 case of varicella, similar to vaccine-associated varicella, which occurs in 10% of normal subjects.
Feldman [14]	MMR and Varicella	281	116	81	9	Safety and Efficacy	8.9 (4.7–13.8)	After final post-transplant vaccines, patients were 92, 83, 94, and 77% seroconverted for measles, mumps, rubella, and varicella. No measles or rubella cases within 1 month post-vaccine. No organ rejection cases within 1 month post-vaccine. One patient with transient non-tender swelling of lymph node, which self-resolved.
Kano [15]	MMR and Varicella	58	7	-	-	Safety and Efficacy	Not available; at least one year	Immunity rates were 100, 100, 90, and 67% for measles, mumps, rubella, and varicella three years after immunization. No side effects were listed for any patients.
Posfay-Barbe [17]	Varicella	77	36	30	7	Safety and Efficacy	3	5 patients developed vesicles, but these remained isolated, and all resolved within 48 h without antiviral therapy. 100% of patients seroconverted after three vaccinations.
Weinberg [18]	Varicella vaccine in patient at least six months outside of liver transplant or 12 months after small bowel transplant	19	19	-	-	Safety and Efficacy	1.1 (0.7–5.6)	4 patients developed rash; three were treated with acyclovir; all lesions healed within 7 days. 13/15 patients seroconverted after 12 weeks post-vaccination.

**Table 2:** Weight-banded standardized dosing for VZIG post-exposure (adapted from VZIG package insert [29]).

Patient’s Weight (in Kilograms)	IU Dose of VZIG
≤2	62.5
2.1–10	125
10.1–20	250
20.1–30	375
30.1–40	500
≥40.1	625
